# Rigid motion‐corrected magnetic resonance fingerprinting

**DOI:** 10.1002/mrm.27448

**Published:** 2018-09-03

**Authors:** Gastão Cruz, Olivier Jaubert, Torben Schneider, Rene M. Botnar, Claudia Prieto

**Affiliations:** ^1^ King’s College London, School of Biomedical Engineering and Imaging Sciences London United Kingdom; ^2^ Philips Healthcare Guilford United Kingdom; ^3^ Pontificia Universidad Católica de Chile, Escuela de Ingeniería Santiago Chile

**Keywords:** low rank, MR fingerprinting, rigid motion correction

## Abstract

**Purpose:**

Develop a method for rigid body motion‐corrected magnetic resonance fingerprinting (MRF).

**Methods:**

MRF has shown some robustness to abrupt motion toward the end of the acquisition. Here, we study the effects of different types of rigid body motion during the acquisition on MRF and propose a novel approach to correct for this motion. The proposed method (MC‐MRF) follows 4 steps: (1) sliding window reconstruction is performed to produce high‐quality auxiliary dynamic images; (2) rotation and translation motion is estimated from the dynamic images by image registration; (3) estimated motion is used to correct acquired k‐space data with corresponding rotations and phase shifts; and (4) motion‐corrected data are reconstructed with low‐rank inversion. MC‐MRF was validated in a standard T_1_/T_2_ phantom and 2D in vivo brain acquisitions in 7 healthy subjects. Additionally, the effect of through‐plane motion in 2D MC‐MRF was investigated.

**Results:**

Simulation results show that motion in MRF can introduce artifacts in T_1_ and T_2_ maps, depending when it occurs. MC‐MRF improved parametric map quality in all phantom and in vivo experiments with in‐plane motion, comparable to the no‐motion ground truth. Reduced parametric map quality, even after motion correction, was observed for acquisitions with through‐plane motion, particularly for smaller structures in T_2_ maps.

**Conclusion:**

Here, a novel method for motion correction in MRF (MC‐MRF) is proposed, which improves parametric map quality and accuracy in comparison to no‐motion correction approaches. Future work will include validation of 3D MC‐MRF to enable also through‐plane motion correction.

## 1 INTRODUCTION

Magnetic resonance fingerprinting (MRF) is a novel relaxometry approach based on continuous sampling of the transient state magnetization evolution.^1^ In MRF, sequence parameters, predominantly flip angle (FA) and repetition time (TR), are varied to explore different magnetization states. Moreover, undersampled trajectories are utilized to sample each combination of sequence parameters (time points) at high temporal resolution. Under these conditions, each unique tissue parameter (e.g., T_1_/ T_2_) combination is expected to produce a unique signal evolution (fingerprint) that can be simulated using Bloch equations or extended phase graphs.^2^, ^3^ The set of simulated signal evolutions (dictionary) can be matched to the measured fingerprints to simultaneously determine tissue parameters like T_1_, T_2_ and *M*_0_. Incoherent spatial and temporal aliasing of the sampled magnetization time points (attributed to undersampling) is typically achieved with non‐Cartesian trajectories to minimize potential bias in dictionary matching step.

Considerable efforts have focused on improving various parts of the MRF approach, among them improving the MRF reconstruction to achieve further scan acceleration. A multiscale MRF reconstruction approach was introduced to reduce the number of measurements by a factor of 3 relative to the original MRF work.^4^ Integration of MRF with simultaneous multislice with an acceleration factor of 2 has also been achieved.^5^ Sliding window and soft‐weighted reconstructions have also been proposed to share data between time points, improving measurement precision and accuracy.^6^, ^7^ Recently, general formulations using low‐rank approximations have been introduced for MRF.^8^, ^9^, ^10^, ^11^, ^12^, ^13^ Low‐rank models have been previously introduced in other MR applications to enable higher acceleration factors.^14^, ^15^, ^16^, ^17^ Fingerprints from different tissues within the dictionary are highly correlated, and methods like singular value decomposition (SVD) can be used to temporally compress fingerprints.^8^ It has been shown that reconstructions in the compressed domain are faster and better posed. A low‐rank projection method operating in k‐space was also proposed, reducing aliasing artifacts.^9^ The low‐rank constraint was directly incorporated into the encoding operator and formulated as a least squares problem in a previous work,^10^ improving parametric map accuracy. A similar approach was taken in another study,^11^ but the reconstruction was further regularized by the best dictionary matches using the alternating direction method of multipliers.^18^

MRF was originally introduced for 2D brain acquisitions.^1^ Recent works have explored further applications, such as abdominal^19^ and cardiac^20^ MRF, where physiological motion is a well‐known problem. Although the original study^1^ demonstrated robustness to abrupt motion toward the end of the acquisition, the impact of motion during the MRF acquisition has not been fully investigated. In conventional MRI, several frameworks have been developed to estimate and correct for motion during the acquisition, particularly for brain applications methods that have been proposed which estimate the motion from the data itself.^21^, ^22^ Some preliminary works have observed sensitivity to motion during the acquisition in MRF.^23^, ^24^, ^25^, ^26^, ^27^ Initial approaches for motion compensation/correction in MRF have also been investigated. In one study,^23^ MRF motion correction was achieved by iteratively identifying corrupted time points, estimating motion, and enforcing data consistency. A different approach^24^ used a sliding window reconstruction, followed by image registration to align the time‐point images before dictionary matching. In a different study,^25^ a similar approach was followed; however, the estimated motion from image registration was used to directly correct the k‐space data and produce a motion‐corrected MRF reconstruction. An alternative approach^26^ introduced gating in MRF as a form of motion compensation. Another approach that has been proposed to minimize motion artifacts is to modify the sampling order such that motion produces incoherent artifacts.^27^

Low‐rank approaches reconstruct compressed images from all acquired data, as mentioned above. In the presence of motion, these reconstructions may introduce motion artifacts (e.g., ghosting and blurring) in addition to quantitative errors attributed to misregistration between time‐point images. In this work, simulations were used to investigate the effect of different types of motion in MRF. A novel approach for rigid motion‐corrected MRF using a low‐rank reconstruction (MC‐MRF) is proposed. With MC‐MRF, rigid body motion is estimated from an auxiliary sliding window reconstruction. The estimated motion is used to correct k‐space, followed by a motion‐corrected low‐rank reconstruction. We investigated the proposed approach to correct for 2D in‐plane motion in a standardized T_1_/ T_2_ phantom and in brain acquisitions in vivo. Additionally, we investigated the effect of through‐plane motion in 2D MC‐MRF.

## 2 METHODS

**Figure 1:**
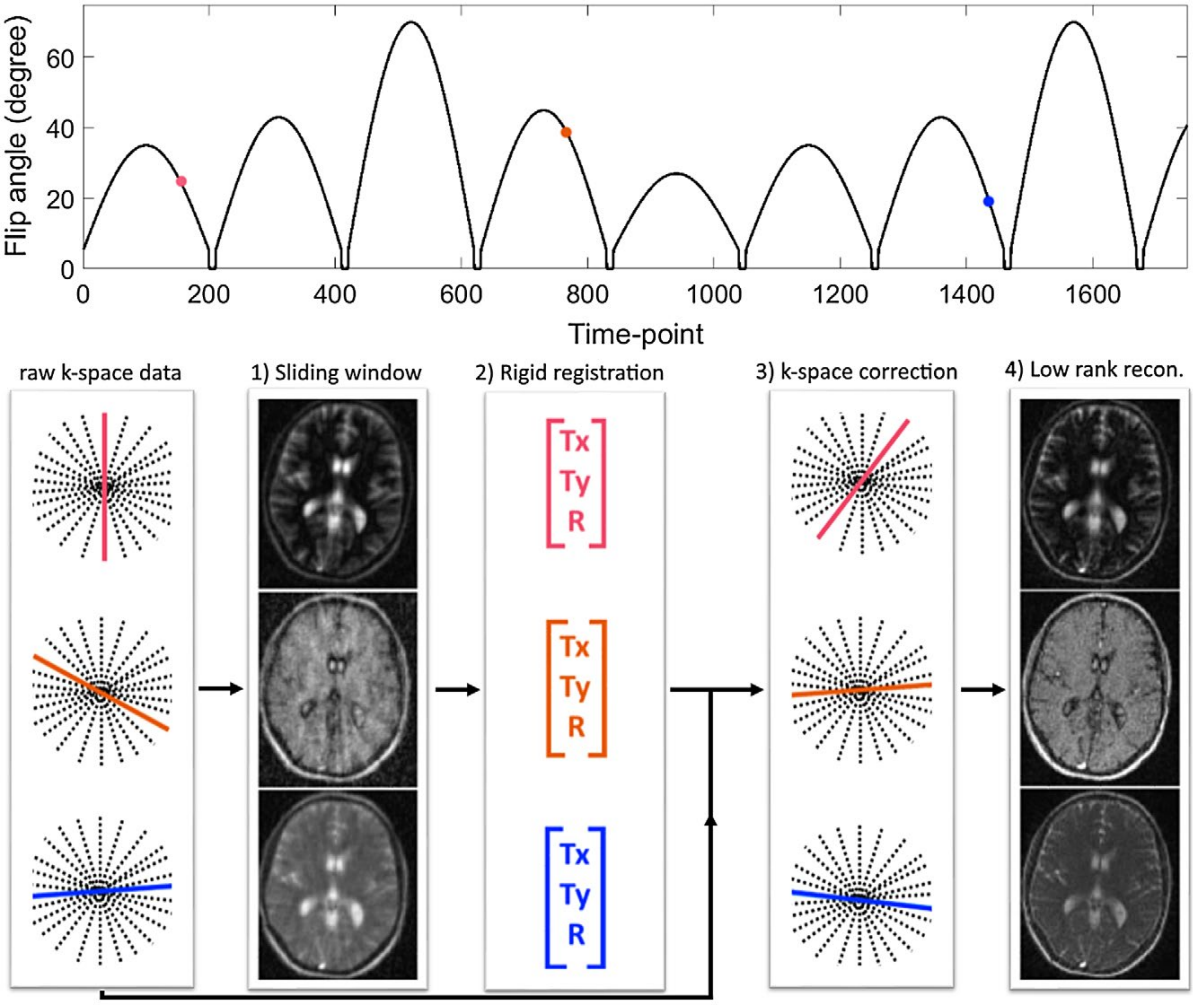
Top: plot of the flip angle pattern used for all experiments, ranging from 0 to 70°. One golden radial spoke was acquired per TR. Bottom: diagram of the proposed MC‐MRF. (1) An iterative SENSE sliding window reconstruction is used to obtain intermediate temporally resolved images with reduced aliasing. (2) Intensity‐based rigid body image registration is used to estimate rotational and 2D translational motion. (3) Rigid motion correction is applied in k‐space with the corresponding phase shifts and rotations. (4) Low‐rank reconstruction of the motion‐corrected k‐space is used to produce the final time points used for matching

The presence of motion during MRF causes a spatial mismatch between time‐point images and causes errors in the estimated maps, as well as ghosting and blurring artifacts, if the data are reconstructed with some data‐sharing techniques (e.g., low‐rank approximation). To address this problem, in this work we propose a novel motion‐corrected MRF reconstruction (MC‐MRF). With the proposed MC‐MRF approach, motion is first estimated and corrected in k‐space preceding a low‐rank reconstruction. The proposed method can be divided in 4 steps: (1) iterative sensitivity encoding (SENSE)^28^ sliding window reconstruction; (2) rigid body image registration; (3) k‐space motion correction; and (4) low‐rank reconstruction.^10^ A diagram of the proposed method is shown in Figure 1. Examples for time‐point images without motion correction and image outputs for steps 1, 2, and 4 of the proposed approach are shown in Supporting Information Figure S1.

### 2.1 Sliding window reconstruction: intermediate images

MRF typically uses highly accelerated image acquisition, where individual time‐point images exhibit severe reconstruction artifacts that can compromise image registration and therefore motion estimation accuracy. Here, an intermediate sliding window reconstruction is used to reduce aliasing artifacts at the expense of temporal resolution. Intermediate images ${\hat{I}}_{t}$ are reconstructed with iterative SENSE (Equation 1):

(1) $${\hat{I}}_{t}=\mathrm{argmi}n_{I_{t}}{∥\left(\mathrm{AFC}I_{t}-K_{t}\right)∥}_{2}^{2}$$

where ***A*** is the sampling operator, ***F*** is the Fourier transform, ***C*** are the coil sensitivities, and $K_{t}$ is the subset of k‐space of a sliding window around time point *t*. Some motion artifacts may be introduced into ${\hat{I}}_{t}$, depending on the motion velocity and size of the sliding window. However, a considerable reduction of aliasing artifacts is expected.

### 2.2 Rigid body image registration

Rigid image registration is applied to the reconstructed intermediate images to estimate translational and rotational motion from each intermediate image to a reference intermediate image. Because of the varying contrast of the intermediate images, registration is performed using mutual information as a similarity measure. To reduce motion estimation errors, image registration is repeated using different reference intermediate images. Estimated motion is taken as the mean of centred motion estimation from each reference.

### 2.3 k‐Space motion correction

The estimated rigid body motion is used to correct the acquired k‐space data K, producing the motion‐corrected $K^{\prime }$. In the presence of rigid motion, the relationship between K and $K^{\prime }$ is given by (Equation 2):

(2) $$K\left(k_{r}\right)=K^{\prime }\left(k_{r}^{\prime }\right)\frac{e^{2\pi i(k_{r}.t)}}{\left|\text{det}(A)\right|}$$

where the matrix ***A*** captures the rigid motion, ***t*** is the translational component of ***A*** and $k_{r}^{\left(\prime \right)}$ are the k‐space coordinates before (after) motion, which are related by $k_{r}^{\prime }=A^{-T}k_{r}$.

### 2.4 Low‐rank reconstruction

The motion‐corrected k‐space $K^{\prime }$ is reconstructed using a low‐rank inversion method.^10^, ^11^ Dictionaries commonly used in MRF are highly compressible along the temporal dimension.^8^ An SVD of the dictionary reveals the first R singular vectors $U_{R}$ (the left singular vectors of the SVD, truncated to rank R), which are incorporated into the encoding operator of the low‐rank reconstruction (Equation 3):

(3) $$\hat{I}=\mathrm{argmi}n_{I}{∥AU_{R}\mathrm{FCI}-K\prime ∥}_{2}^{2}$$

where ***I*** are the singular value images, related to the time‐point images $I^{\prime }$ by $I=U_{R}^{H}I^{\prime }$. Every singular image is a linear combination of data from (potentially) every time point. Thus, if motion is not accounted for, this reconstruction will create typical motion artifacts (e.g., ghosting and blurring), in addition to misregistration between time‐point images. Here, k‐space is motion corrected before low‐rank reconstruction, eliminating these issues.

### 2.5 Experiments

The effects of motion in MRF and the proposed motion correction approach were validated in simulations and a phantom moved continuously by hand. The proposed approach was evaluated with in vivo 2D brain scans in the presence of in‐plane, through‐plane motion and no motion.

Phantom and in vivo brain data were acquired on a 1.5 Tesla Ingenia MR system (Philips, Best, The Netherlands) using a 12‐element head coil. The study was approved by the institutional review board, and written informed consent was obtained from all subjects according to institutional guidelines.

### 2.6 Simulations

A digital phantom modeled after a brain scan with realistic T_1_, T_2_, and M_0 _values was used to evaluate the effects of motion in MRF. Acquisition was performed with similar parameters to Jiang et al. using the same modifications as for the in vivo acquisitions, described below. Three motion simulations were performed by modifying the acquired motion free k‐space: (1) 2D abrupt rigid motion occurring at time point #250 (out of 1750 time points, toward the beginning of the acquisition); (2) 2D abrupt rigid motion occurring at time point #1500 (out of 1750 time points, toward the end of the acquisition); and (3) 2D continuous sinusoidally varying rigid motion. Simulations (1) and (2) used motion amplitudes of *T*_x_ = 8 pixels (left‐right translation), *T*_y_ = 2 pixels (anterior‐posterior translation), and *R* = 12º (in‐plane rotation). Simulation (3) used motion amplitudes of *T*_x_ = 8 pixels, *T*_y_ = 2 pixels, and *R* = 24º. The proposed MC‐MRF was compared with an image‐based motion correction (IBMC) similar to the approach proposed in a previous work.^24^ In IBMC, time‐point images are reconstructed using sliding window reconstruction; these images are then registered to a common motion state and summed before performing dictionary matching. Here, IBMC was implemented considering the first time point of the sequence as the reference for motion alignment.

### 2.7 Data acquisition

2D single‐slice acquisitions were performed on a standardized T_1_/ T_2_ phantom^29^ and in 7 healthy subjects. A similar protocol to another work^30^ was used with FA varying from 0 to 70°, however, with the following differences: FA pattern was slightly modified (Figure 1), and constant TR and golden radial trajectory^31^ were used. Relevant acquisition parameters are as follows: gradient echo readout, fixed echo time/TR = 1.23/4.3 ms, 1750 time points, 2 × 2 mm^2^ in‐plane resolution, 10‐mm slice thickness, 320 × 320 mm^2^ field of view (FOV), transverse slice, 1 golden radial line per time point, and 160 points per radial spoke. An inversion recovery (IR) pulse was applied before the beginning of the acquisition. In the phantom experiment, the phantom was continuously moved by hand in the direction perpendicular to the table throughout the acquisition (left‐right direction in the corresponding FOV). For the brain acquisitions, subjects were instructed for 3 scans: (1) no head motion, (2) continuous (mostly) in‐plane motion (yaw), and (3) continuous (mostly) through‐plane motion (roll).

### 2.8 Image reconstruction

The proposed MC‐MRF method was implemented offline in MATLAB (The MathWorks, Inc, Natick, MA). Coil sensitivity maps were estimated from the data itself using ESPIRiT,^32^ density compensation functions were computed by Voronoi diagrams,^33^ and nonuniform Fourier transform based on a previous work^34^ was used. All reconstructions were solved with the conjugate gradient method, with maximum number of iterations set to 15 (chosen to naturally regularize the solution). The intermediate sliding window reconstruction used a window of 50 time points (corresponding to a temporal window of ~200 ms). The number of time points per window used was determined by inspecting the image quality of several sliding window reconstructions in simulations. This amount of data sharing minimizes aliasing artifacts (which may compromise motion estimation) while maintaining sufficient temporal resolution for head motion estimation. Image registration was performed with MATLAB’s image processing toolbox using normalized mutual information as a metric and a gradient descent optimizer. Motion was estimated by registering each intermediate image toward a given reference. To minimize motion estimation errors attributed to varying signal intensity throughout the MRF acquisition, the registration was repeated multiple times using different intermediate images as a reference. Registrations with different references were performed in parallel. The set of reference images was given by 1:100:N_t_, where N_t_ is the total number of time points. The distance of 100 intermediate images was found empirically adequate to minimize motion estimation errors. Motion parameters were estimated by registration to each reference intermediate image. Subsequently, each estimated motion parameter was centered by subtracting its mean, putting all the estimated parameters in a common frame of reference. Finally, the estimated motion was taken as the average of the centered motions obtained from the registration to the different references. Translational and rotational motions were corrected applying the corresponding phase shifts and k‐space rotations as described in Equation (2). The low‐rank approximation was experimentally determined to have a rank *R* = 10. The proposed method took approximately 120 minutes (approximately 90 minutes for the sliding window reconstruction, 20 minutes for rigid registration, and 10 minutes for low‐rank reconstruction) on a Linux workstation with 12 Intel Xeon X5675 (3.07 GHz) and 200 GB RAM. Low‐rank reconstruction, with the same parameters that were described before, was also utilized to reconstruct data without motion correction for both the motion‐corrupted and motion‐free acquisitions. Data and MATLAB code of the proposed approach are available at https://kclcvmimaging.wordpress.com/downloads/.

### 2.9 Dictionary and pattern recognition

The MRF dictionaries were simulated using the extended phase graph formalism,^2^, ^3^ based on code available.^3^ Slice profile correction was utilized^35^; however, no B_1_ correction was used. Template matching between fingerprints and dictionary were performed using the inner product.^30^ Separate dictionaries were used for the phantom and brain data sets based on the expected tissue range. For the phantom data, T_1_ ∈ [0:30:200, 200:10:600, 600:20:1200, 1200:30:1600] ms, T_2_ ∈ [0:2:70, 70:10:120, 120:5:270] ms; for the brain data, T_1_ ∈ [0:10:800, 800:40:1400, 1400:300:6000] ms, T_2_ ∈ [0:5:100, 100:10:500, 500:50:1000, 1000:300:2600] ms. For the slice profile correction, the slice was discretized into 50 points along the frequency dimension.

## 3 RESULTS

### 3.1 Simulations

**Figure 2:**
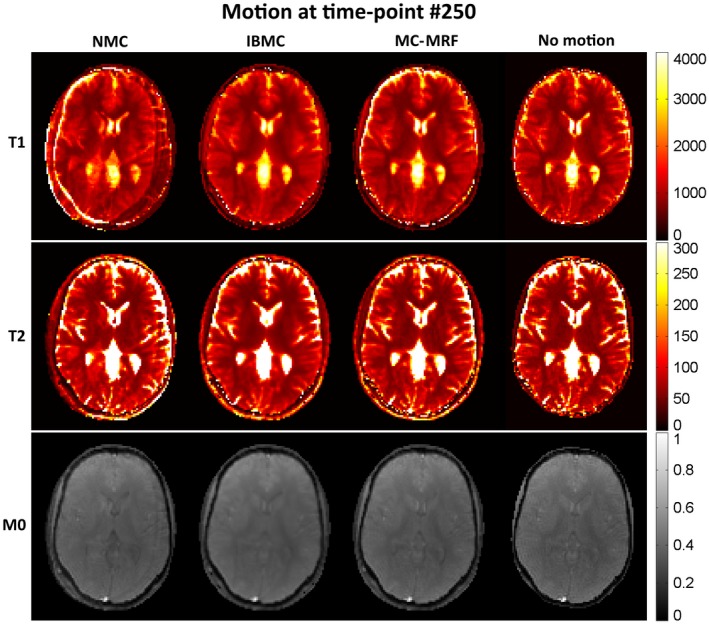
MRF simulations with no motion correction (NMC), IBMC, and the proposed MC‐MRF for abrupt rigid motion at time‐point 250 (out of 1750 time points). Motion toward the beginning of acquisition affects primarily *T*_1_, but residual ghosting artifacts are also present in the *T*_2_ and *M*_0_ maps. MC‐MRF reduces most motion artifacts and achieves similar image quality than the motion‐free reference. IBMC also achieves good motion correction; however, residual blurring artifacts are present. Estimated motion parameters for this simulation are shown in the corresponding Supporting Information Figure S2

**Figure 3:**
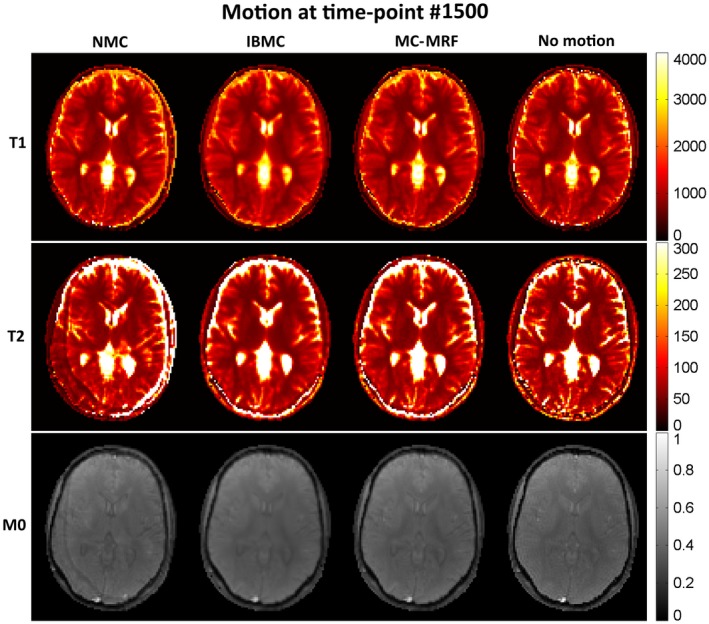
MRF simulations with no motion correction (NMC), IBMC, and the proposed MC‐MRF for abrupt rigid motion at time‐point 1500 (out of 1750 time points). Motion toward the end of acquisition affects primarily *T*_2_ (and M_0_), but residual ghosting artifacts are also present in the *T*_1_ map. MC‐MRF reduces most motion artifacts and achieves similar image quality than the motion‐free reference. IBMC also achieves good motion correction; however, residual blurring artifacts are present. Estimated motion parameters for this simulation are shown in the corresponding Supporting Information Figure S3

**Figure 4:**
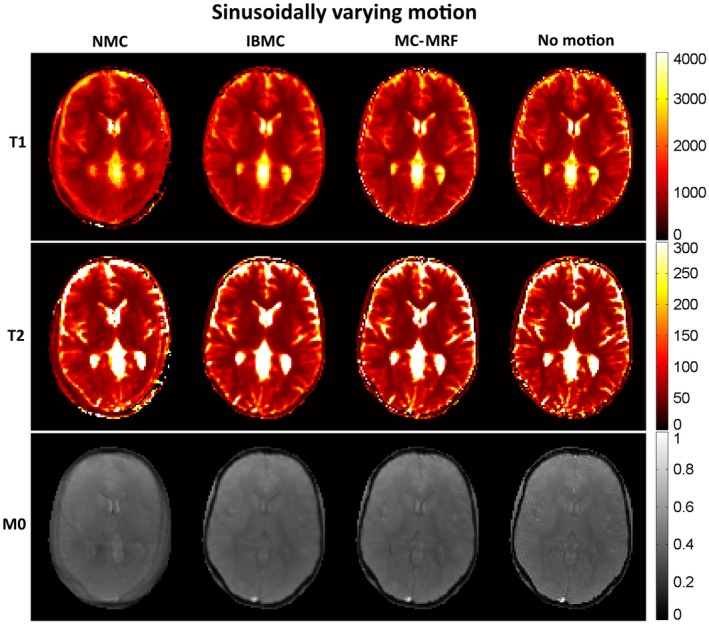
MRF simulations with no motion correction (NMC), IBMC, and the proposed MC‐MRF for sinusoidally varying motion. All parametric maps are affected by continuous motion. MC‐MRF reduces most motion artifacts and achieves similar image quality than the motion‐free reference. IBMC also achieves good motion correction; however, residual blurring artifacts are present. Estimated motion parameters for this simulation are shown in the corresponding Supporting Information Figure S4

T_1_, T_2_, and *M*_0_ maps from MRF reconstruction without motion correction, with IBMC and with the proposed MC‐MRF, are shown in Figures 2, 3, and 4 for the 3 different types of rigid motion simulated, respectively: (1) 2D abrupt motion at time‐point #250 (toward the beginning of the acquisition); (2) 2D abrupt motion at time‐point #1500 (toward the end of the acquisition); and (3) 2D sinusoidally varying motion. In the first case (Figure 2), abrupt motion occurs during high encoding of T_1_ (close to the IR pulse) and affects primarily the T_1_ map. In the second case (Figure 3), abrupt motion occurs near high encoding of T_2_ (high FA) and has corresponding effects in T_2_. In the third case (Figure 4), sinusoidal motion affects all time points, and corresponding blurring and ghosting artifacts appear in both T_1_ and T_2_ maps. Motion artifacts in *M*_0_ appear more correlated with motion artifacts in T_2_ than in T_1_. With MC‐MRF, motion artifacts were virtually eliminated in comparison to the motion‐free gold standard; however, a slight reduction in resolution was also observed. Rotational motion disturbs the quasi‐uniform golden‐angle distribution, opening gaps in k‐space. This effect makes the following low‐rank reconstruction more ill‐conditioned and can produce residual artifacts. With the IBMC, improvements in the parametric maps were also achieved; however, residual blurring artifacts were also present, leading to a further decrease in apparent resolution and increase in apparent signal‐to‐noise ratio (SNR). This is attributed to interpolation effects, residual uncorrected motion within each sliding window, and residual errors in motion estimation.

Corresponding estimated translation and rotation motion for IBMC and MC‐MRF are shown in Supporting Information Figures S2, S3, and S4 for the 3 different types of motion, respectively. IBMC produced motion estimates similar to MC‐MRF, albeit with slightly more errors. This is attributed to IBMC estimating motion from a single reference image registration, as opposed to using multiple references as in MC‐MRF. Good accuracy was generally achieved with MC‐MRF; however, small errors in motion estimated were observed around time points of high velocity motion (abrupt discontinuities). Indeed, because MC‐MRF estimates motion from intermediate images with a temporal resolution of ~200 ms, it fails to capture abrupt motion in the order of the TR (ie, ~4 ms).

### 3.2 Phantom acquisition

**Figure 5:**
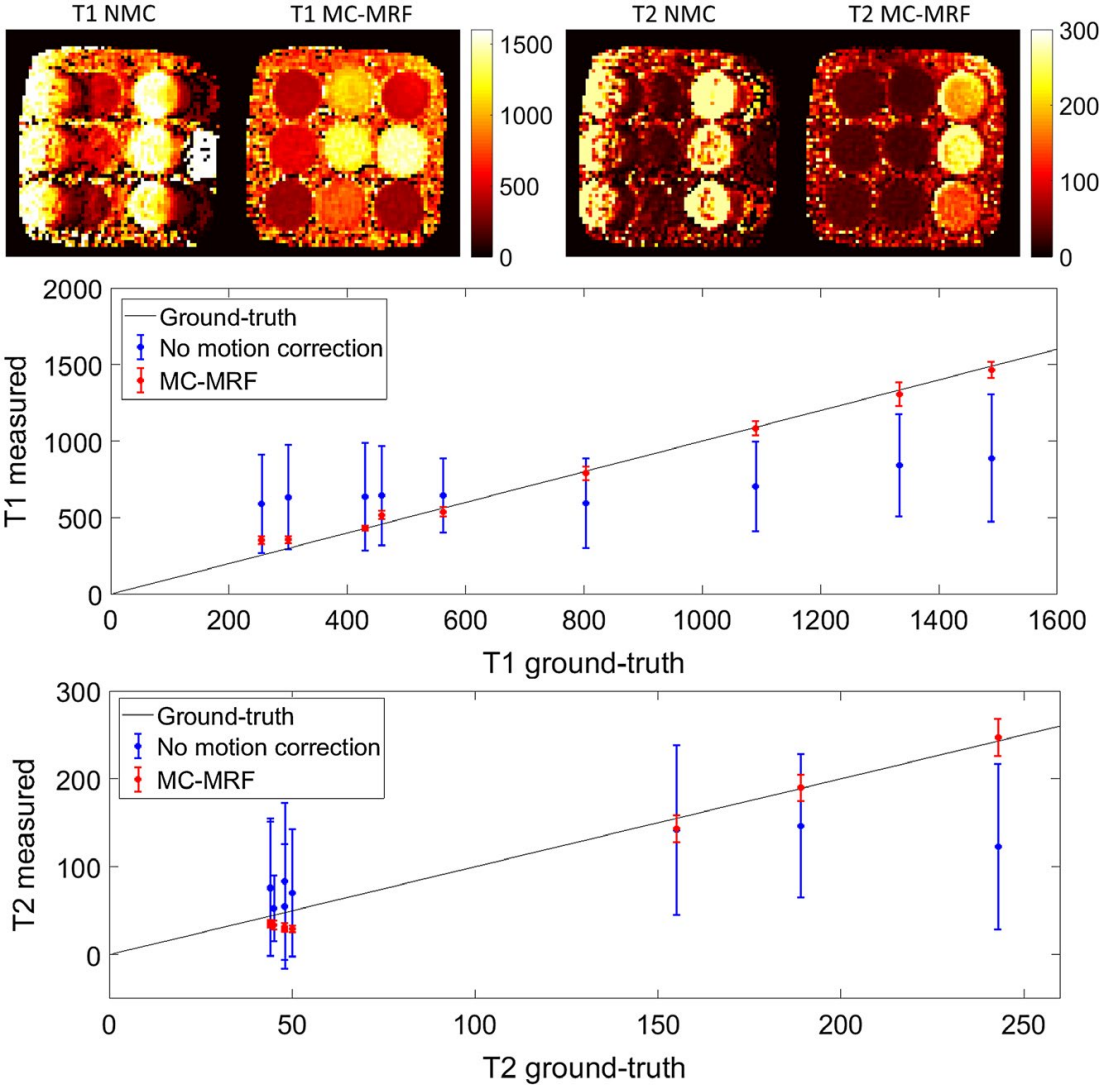
Results for a manually moved phantom experiment with predominant translational motion and minimal rotational motion. Considerable motion artifacts can be observed in both *T*_1_ and *T*_2_ maps in the case of no motion correction (NMC). These artifacts are greatly reduced with the proposed MC‐MRF. Plots for the *T*_1_ and *T*_2_ values of the parametric phantom are shown below in comparison to gold‐standard values, where MC‐MRF considerably improves the accuracy and precision of the measurements

Parametric maps for the phantom experiment are shown in Figure 5 (top). Considerable motion artifacts propagate into the parametric maps without motion correction. With the proposed MC‐MRF approach, the motion artifacts are virtually eliminated. Corresponding plots for T_1_ and T_2_ in comparison to gold‐standard values^29^ are shown in Figure 5 (bottom). A loss in precision and accuracy occurs without motion correction; improvements in both these metrics are achieved with the proposed MC‐MRF motion correction.

### 3.3 In vivo brain acquisitions

**Figure 6:**
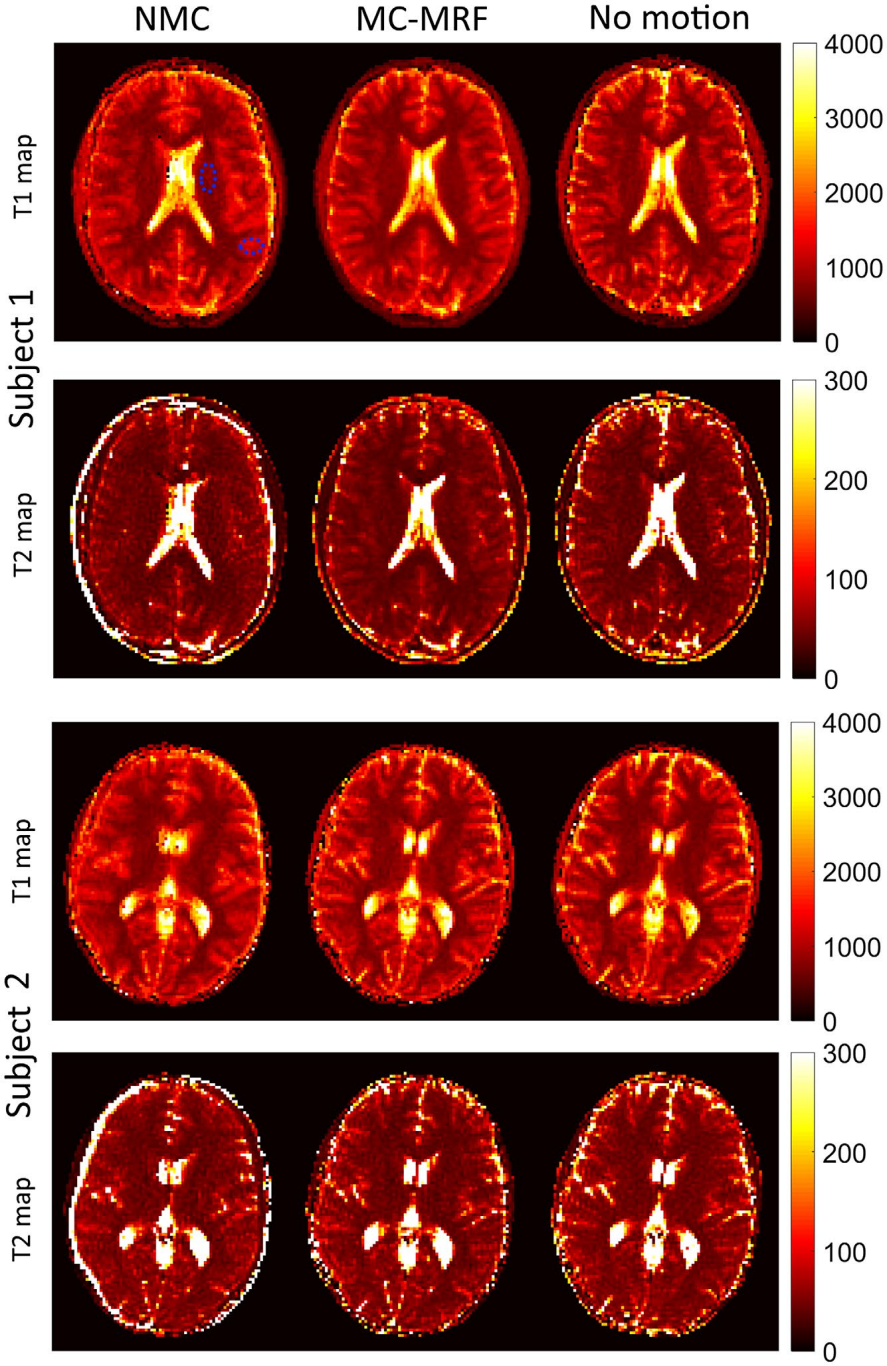
In vivo results with in‐plane motion for subjects 1 and 2 with no motion correction (NMC), MC‐MRF, and no motion. Ghosting and blurring artifacts propagate from the time‐point images into the parametric maps in the NMC case. The proposed MC‐MRF improves parametric map quality, to a comparable degree to the case of no motion. Dotted blue areas in NMC T_1_ for subject 1 denote the regions of interest used to measure white and gray matter parametric values. Estimated motion parameters for these subjects are shown in the Supporting Information Figure S6

**Figure 7:**
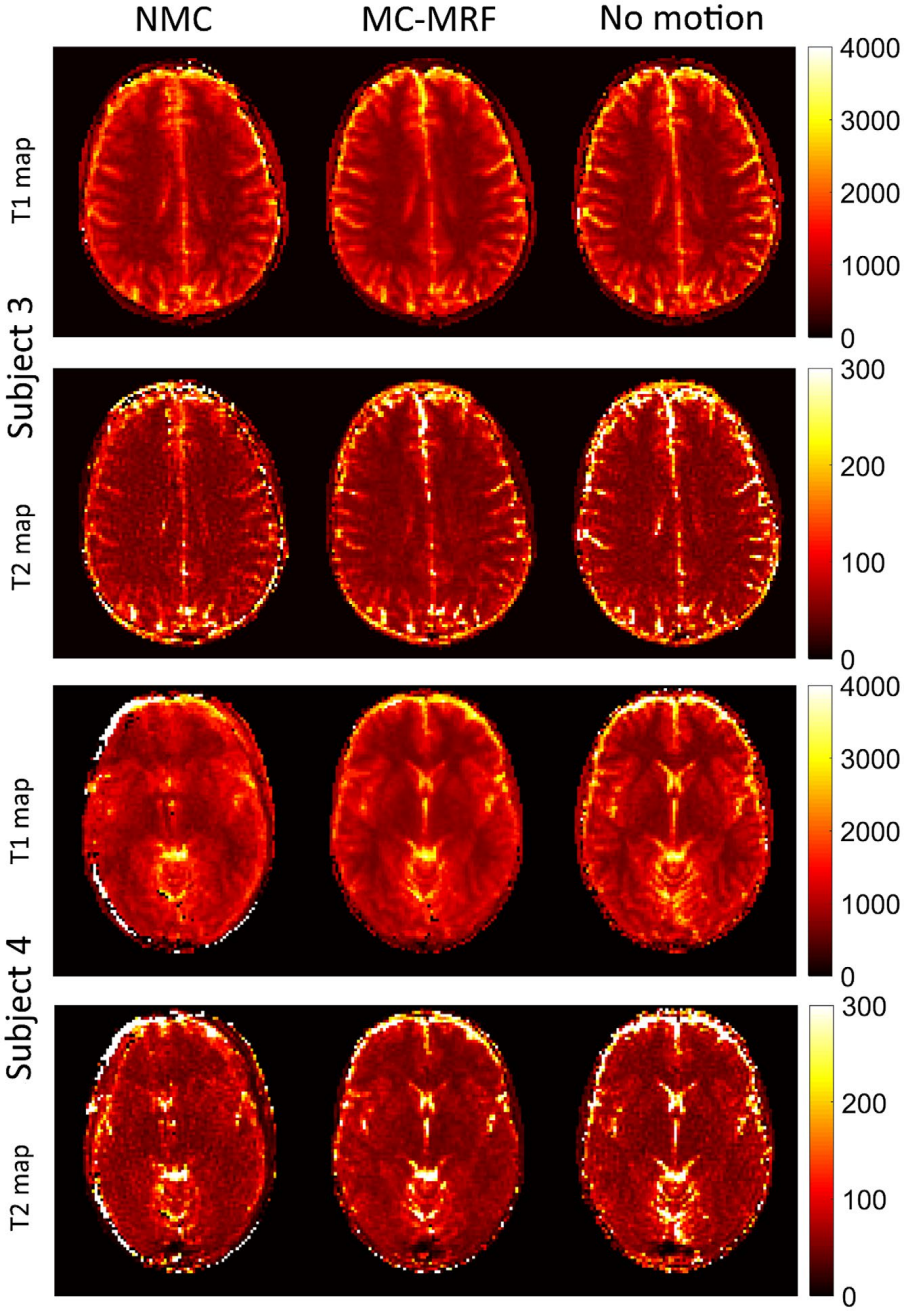
In vivo results with in‐plane motion for subjects 3 and 4 with no motion correction (NMC), MC‐MRF, and no motion. Ghosting and blurring artifacts propagate from the time‐point images into the parametric maps in the NMC case. The proposed MC‐MRF improves parametric map quality, to a comparable degree to the case of no motion. Estimated motion parameters for these subjects are shown in the Supporting Information Figure S6

Selected time‐point images with and without in‐plane motion correction are shown in Supporting Information Figure S5, for 2 subjects (1 and 2). Blurring and ghosting artifacts (in addition to misregistration) are visible when no motion correction is used; conversely, both these effects are minimized with the proposed MC‐MRF approach. T_1_, T_2_, and *M*_0_ maps are shown for 4 representative subjects in Figures 6 and 7. Results without motion correction have ghosting and blurring artifacts, obscuring several brain structures. Motion correction improves parametric maps to a similar quality of the case without motion. The estimated in‐plane motion amplitudes for rotation, left‐right translation, and anterior‐posterior translation in the format [minimum, average, maximum] were *R* = [5.6, 11.1, 18.3]°, T_x_ = [5.2, 9.3, 19.4] mm, and T_y_ = [0.4, 1.1, 1.8] mm, respectively. The corresponding estimated amplitudes for the through‐plane experiments were *R* = [3.1, 9.3 22.1]°, T_x_ = [8.7, 17.1, 32.2] mm, and T_y_ = [0.6, 1.8, 4.4] mm, respectively. Subject 1 had the minimum estimated motion amplitudes, whereas subject 4 had the maximum estimated motion amplitudes. The estimated in‐plane motion plots in Supporting Information Figure S6 capture the continuous cyclical motion performed by the subjects in Figures 6 and 7.

**Figure 8:**
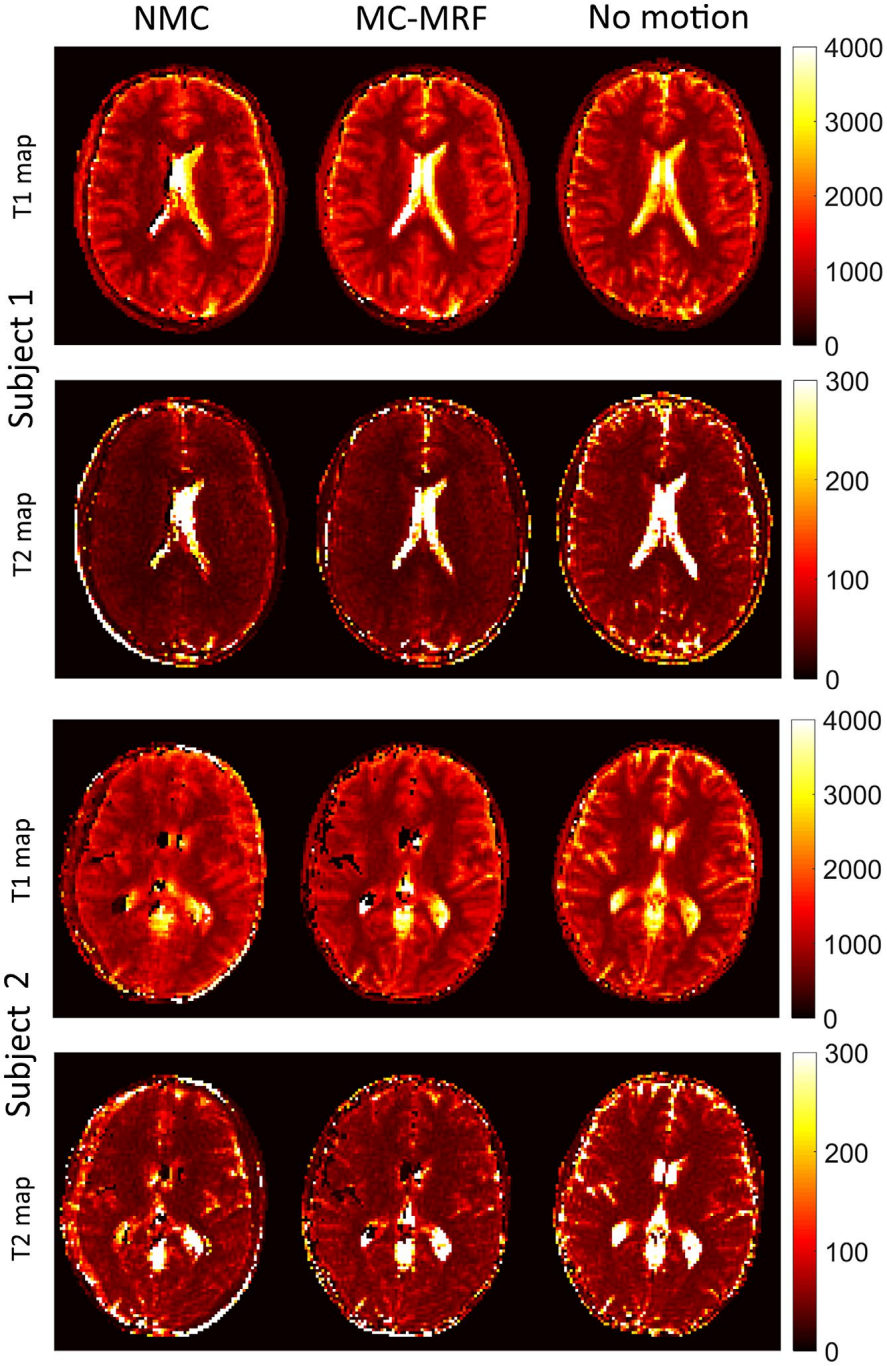
In vivo results with through‐plane motion for subjects 1 and 2 with no motion correction (NMC), MC‐MRF, and no motion. Considerable motion artifacts are present in NMC. Artifacts are reduced with the proposed MC‐MRF; however, residual errors remain in the parametric maps, especially in T_2_

**Figure 9:**
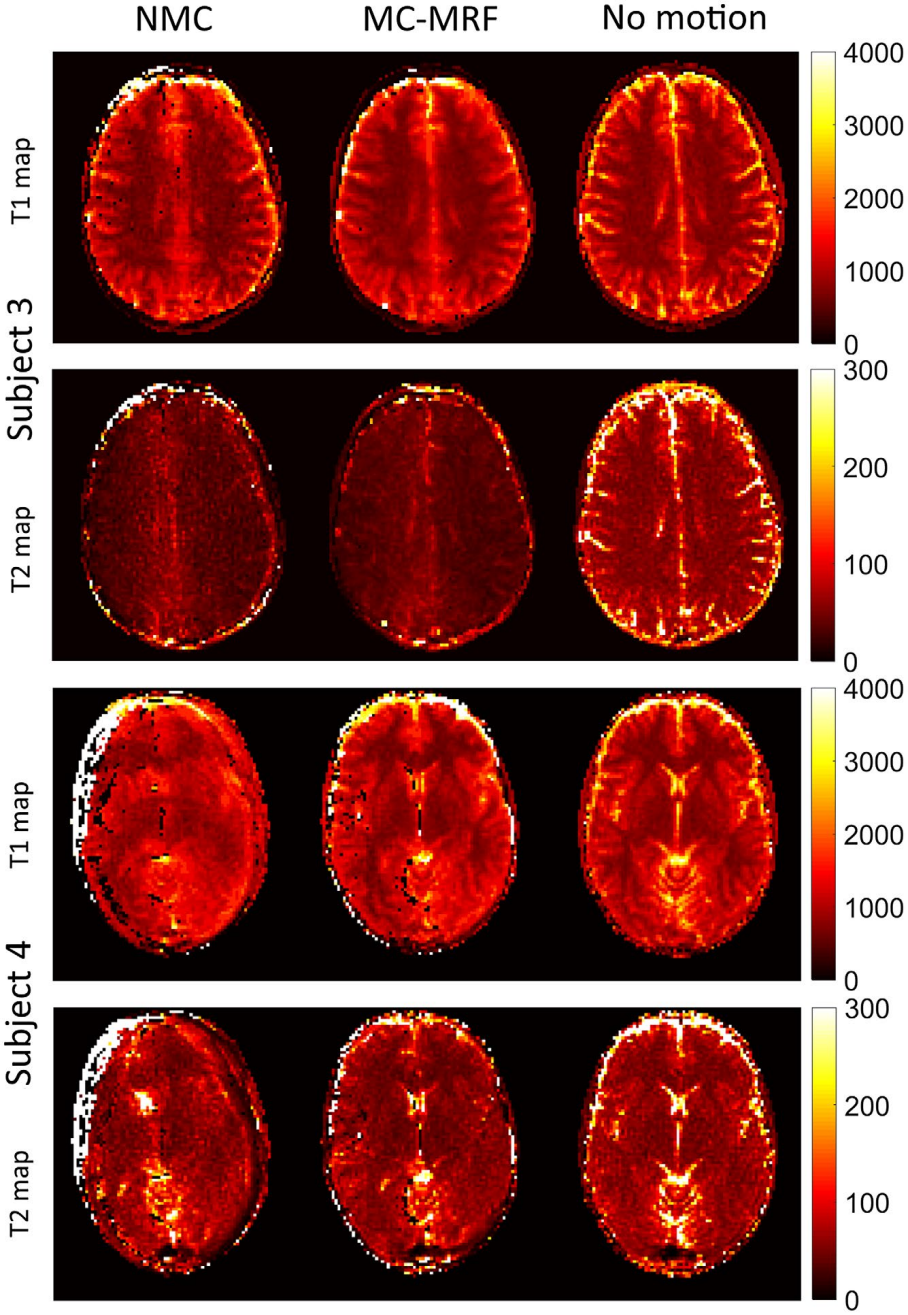
In vivo results with through‐plane motion for subjects 3 and 4 with no motion correction (NMC), MC‐MRF, and no motion. Considrable motion artifacts are present in NMC. Artifacts are reduced with the proposed MC‐MRF; however, residual errors remain in the parametric maps, especially in *T*_2_

T_1_, T_2_, and *M*_0_ maps for the case of through‐plane motion are shown for the same 4 representative subjects in Figures 8 and 9. Again, motion artifacts are present without motion correction. The proposed MC‐MRF corrects for some of this motion; however, considerable artifacts remain after motion correction. These residual artifacts from through‐plane motion appear predominantly on the left and right sides of the brain (where maximum through‐plane rotation occurs) and have a stronger impact on the T_2_ maps.

T_1_ and T_2_ values for several regions of interest (denoted in Figure 6) are shown in Table 1. T_1_ values agreed with literature; however, T_2_ values were underestimated for white and gray matter. An additional reduction in observed T_2_ occurred for cases of through‐plane. Parametric values for MC‐MRF in the presence of in‐plane motion were comparable with the case of no motion; considerably higher standard deviation was observed for the cases of through‐plane motion.

**Table 1:** *T*_1_ and *T*_2_ in healthy subjects for no motion correction (NMC), the proposed MC‐MRF, and the ground truth (no motion)

	**In‐plane** ** NMC**	**In‐plane ** **MC‐MRF**	**Through‐plane** **NMC**	**Through‐plane** **MC‐MRF**	**No motion**	**Literature **
T1 white matter	753 ± 33	743 ± 32	769 ± 58	721 ± 47	738 ± 20	608–756
T2 white matter	49 ± 6	47 ± 5	41 ± 7	39 ± 6	48 ± 5	54–81
T1 gray matter	1074 ± 22	1159 ± 29	1069 ± 64	1104 ± 61	1127 ± 28	998–1304
T2 gray matter	63 ± 5	63 ± 4	47 ± 9	47 ± 9	69 ± 5	78–98

## 4 DISCUSSION

A novel method for rigid body motion correction in MRF was proposed and validated in simulations, a standardized phantom, and brain data of healthy subjects. The proposed approach estimates motion from an intermediate sliding window reconstruction (by image registration) and corrects k‐space before a low‐rank (motion corrected) reconstruction. The framework does not require additional training data and is suitable for accelerated MRF because of the low‐rank reconstruction. The proposed motion correction method successfully improved parametric maps to a comparable degree to that of no motion for in‐plane motion. As expected, residual errors for through‐plane motion remained after motion correction, especially in T_2_ maps.

Simulations show that motion in MRF can affect both T_1_ and T_2_ maps, depending when it happens in the acquisition. Given that the T_1_/ T_2_ encoding power varies during the acquisition, motion at different time points will corrupt the parametric maps differently. Most MRF sequences rely on an initial inversion recovery pulse to encode T_1_; consequently, motion toward the beginning of the acquisition affects primarily the T_1_ map. T_2_ encoding in MRF is generally achieved with spin and stimulated echoes (proportional to sin^2^ (*FA*/2) and sin (FA), respectively); consequently, time points associated with echo creation are more likely to affect the T_2_ map. Motion will affect misregistration between time points and introduces motion artifacts if the time‐point images are reconstructed from k‐space data acquired at multiple motion states (e.g., low‐rank approximation). Misregistration will cause each pixel’s tissue to change during the acquisition, effectively making T_1_ and T_2_ vary with time. Consequently, misregistration will cause the most errors in the border between tissues where the fingerprint will oscillate between different T_1_/ T_2_ values during the acquisition. This bias can affect the template matching step in MRF, leading to a mismatch in the dictionary. Motion artifacts are generally split into blurring and ghosting. Blurring artifacts will give pixels a mix of signal from different tissues. The fingerprint will correspond to a combination of different T_1_/ T_2_, similar to a partial volume problem. Ghosting artifacts behave like undersampling artifacts. Temporally, these artifacts are also expected to be noise‐like; in the presence of considerable artifacts, the fingerprint template match may also fail because of excessive noise.

The proposed MC‐MRF was compared with an alternative IBMC in simulations. Parametric maps obtained with IBMC achieved comparable quality to MC‐MRF; however, IBMC suffered from residual blurring artifacts. At higher acceleration factors, IBMC is expected to produce more aliasing artifacts than MC‐MRF. Additionally, if the motion approaches the temporal resolution of the sliding window, residual motion artifacts will propagate into the parametric maps of the IBMC, whereas MC‐MRF can correct for motion within the sliding window temporal resolution. A comparison between alternative strategies for MRF motion correction will be of interest in future work.

For the in‐plane in vivo acquisitions MC‐MRF provides T_1_ and T_2_ white and gray matter values comparable to those obtained from a motionless scan. White and gray matter T_1_ values were in good agreement with those reported in literature; however, T_2_ values were underestimated. Errors in T_2_ with respect to literature values were also observed for the no‐motion acquisitions. Reduced acquisition time led to a reduction of T_2_ encoding with the current FA pattern. Different FAs patterns that have been shown to be more sensitive to T_2_ 36 in short acquisition times will be investigated in future work. Here, *B*_1_ was not corrected for, which could also lead to errors in T_2_.

One of the main limitations of the proposed study is its validation in 2D acquisitions, which cannot account for through‐plane motion. Results showed that considerable errors remain, especially underestimation of T_2_, even after using the proposed MC‐MRF to correct for in‐plane motion. Given that different tissues enter and leave the slice, the effective T_1_/ T_2_ would be described by a time‐varying partial volume model. Additionally, because a given tissue moves within the slice, it will experience a different *B*_1_ phase and amplitude, altering the magnetization history of that tissue. This effect compromises the fingerprint and, consequently, the template matching. If through‐plane motion could be estimated (in relation to measured in‐plane motion perhaps), the effect could be corrected by modeling a temporally varying *B*_1_ for each spatial location along the slice profile, although it would be computationally expensive. Prospective motion correction could also be used, although other challenges would have to be considered.^37^ A slice thickness of 10 mm was used in these experiments to avoid low SNR and to help reduce accidental through‐plane motion during in‐plane motion experiments. This way, the effects of in‐plane and through‐plane motion in MRF could be better separated. Future work will consider higher in‐ and through‐plane resolutions.

The natural solution for through‐plane motion will generally be 3D acquisitions. 3D radial trajectories will be considered^38^, ^39^ to extend the proposed approach. Adequate temporal resolution for the sliding window reconstruction may be a challenge in 3D. In this case, spatial resolution of the sliding window may be reduced or additional regularization may be used (e.g., compressed sensing).^40^ Additionally, the FOV and/or the TR may need to be reduced. Another limitation of the current work is the temporal resolution of the estimated motion (~200 ms). This resolution is reasonable for head motion or even respiratory motion, but would not be sufficient for faster motion (e.g., cardiac). Alternatively, motion estimation could be achieved by autofocus,^41^ potentially achieving a temporal resolution of the order of the TR.

The proposed method only corrects for rigid body motion. Motion correction becomes more challenging as the motion amplitude increases because of possible motion estimation inaccuracies, larger k‐space gaps, and, possibly, higher degree of through‐plane motion. Expanding the motion correction to more complete models (affine and nonlinear) is also of interest in future work. Whereas extension to affine motion correction would be straightforward (using Equation 2), general elastic motion would require more complex solutions such as a motion compensated reconstruction^42^ or localized autofocus.^43^ Finally, the current suboptimal implementation of the proposed framework features slow reconstruction times. This can be improved by reconstructing only a subset of sliding window time points for motion estimation (with adequate temporal resolution) and/or by reconstructing lower spatial resolution images (which should be sufficient for rigid motion estimation, but may be not be the case for more complex models such as affine or elastic motion).

Future work should incorporate effects missing in the current model, such as B_1_ inhomogeneity^44^ or magnetization transfer effects.^45^ B_0_, in addition to being able to both transmit and receive B_1_ fields, can vary in the presence of motion and may need to be accounted for in the dictionary simulation. Extension of the current method to 3D MRF^46^ is desirable; through‐plane motion will be eliminated and motion correction will be more relevant because of the increased scan time. Finally, more complex motion models will be required if the method is to be considered for abdominal or cardiac MRF.

## 5 CONCLUSION

A novel method for rigid body motion correction in MRF has been proposed and validated in vivo for 2D acquisitions. For in‐plane motion, the proposed motion correction approach produces similar T_1_ and T_2_ maps to the case of no motion; however, residual errors exist in the case of through‐plane motion, particularly for T_2_.

## Supporting information


**FIGURE S1** Examples of time‐point images for a low‐rank reconstruction with no motion correction (non‐motion‐corrected) and intermediate time points at different stages of the proposed framework: (1) sliding window reconstruction, (2) rigid registration, and (4) (motion corrected) low‐rank reconstruction.
**FIGURE S2** Estimated motion parameters for the simulation experiment with abrupt rigid motion occurring at time point 250, using IBMC and the proposed MC‐MRF. Generally, both methods achieve accurate motion estimation; however, higher errors are present for IBMC. Both methods present motion estimation errors around the abrupt motion discontinuities.
**FIGURE S3** Estimated motion parameters for the simulation experiment with abrupt rigid motion occurring at time point 1500, using IBMC and the proposed MC‐MRF. Generally, both methods achieve accurate motion estimation; however, higher errors are present for IBMC. Both methods present motion estimation errors around the abrupt motion discontinuities.
**FIGURE S4** Estimated motion parameters for the simulation experiment with sinusoidally varying motion, using IBMC and the proposed MC‐MRF. Generally, both methods achieve accurate motion estimation; however, higher errors are present for IBMC.
**FIGURE S5** Time‐point images for subjects 1 and 2 with no motion correction (NMC) and the proposed MC‐MRF from an acquisition with in‐plane motion. In the presence of motion, low‐rank reconstruction with no motion correction introduces ghosting and blurring. MC‐MRF greatly reduces motion artefacts, revealing image structures otherwise obscured.
**FIGURE S6** Estimated rigid body motion in 4 representative brain subject in vivo scans with in‐plane motion. Rotational motion is shown in blue, left‐right translation is shown in continuous red, and anterior‐posterior translation is shown in dashed red. The estimated motion captures the periodic nature of motion in subjects instructed to continuously move during the acquisition.
