# Supplementary material for: Rigid motion‐corrected magnetic resonance fingerprinting
Source: Magn Reson Med. 2018 Sep 3;81(2):947–61. doi: 10.1002/mrm.27448 (PMC6519164; doi:10.1002/mrm.27448)
Supplement: Supplementary file 1 — FIGURE S1 Examples of time‐point images for a low‐rank reconstruction with no motion correction (non‐motion‐corrected) and intermediate time points at different stages of the proposed framework: (1) sliding window reconstruction, (2) rigid registration, and (4) (motion corrected) low‐rank reconstruction. FIGURE S2 Estimated motion parameters for the simulation experiment with abrupt rigid motion occurring at time point 250, using IBMC and the proposed MC‐MRF. Generally, both methods achieve accurate motion estimation; however, higher errors are present for IBMC. Both methods present motion estimation errors around the abrupt motion discontinuities. FIGURE S3 Estimated motion parameters for the simulation experiment with abrupt rigid motion occurring at time point 1500, using IBMC and the proposed MC‐MRF. Generally, both methods achieve accurate motion estimation; however, higher errors are present for IBMC. Both methods present motion estimation errors around the abrupt motion discontinuities. FIGURE S4 Estimated motion parameters for the simulation experiment with sinusoidally varying motion, using IBMC and the proposed MC‐MRF. Generally, both methods achieve accurate motion estimation; however, higher errors are present for IBMC. FIGURE S5 Time‐point images for subjects 1 and 2 with no motion correction (NMC) and the proposed MC‐MRF from an acquisition with in‐plane motion. In the presence of motion, low‐rank reconstruction with no motion correction introduces ghosting and blurring. MC‐MRF greatly reduces motion artefacts, revealing image structures otherwise obscured. FIGURE S6 Estimated rigid body motion in 4 representative brain subject in vivo scans with in‐plane motion. Rotational motion is shown in blue, left‐right translation is shown in continuous red, and anterior‐posterior translation is shown in dashed red. The estimated motion captures the periodic nature of motion in subjects instructed to continuously move during the acquisition. [file MRM-81-947-s001.docx]

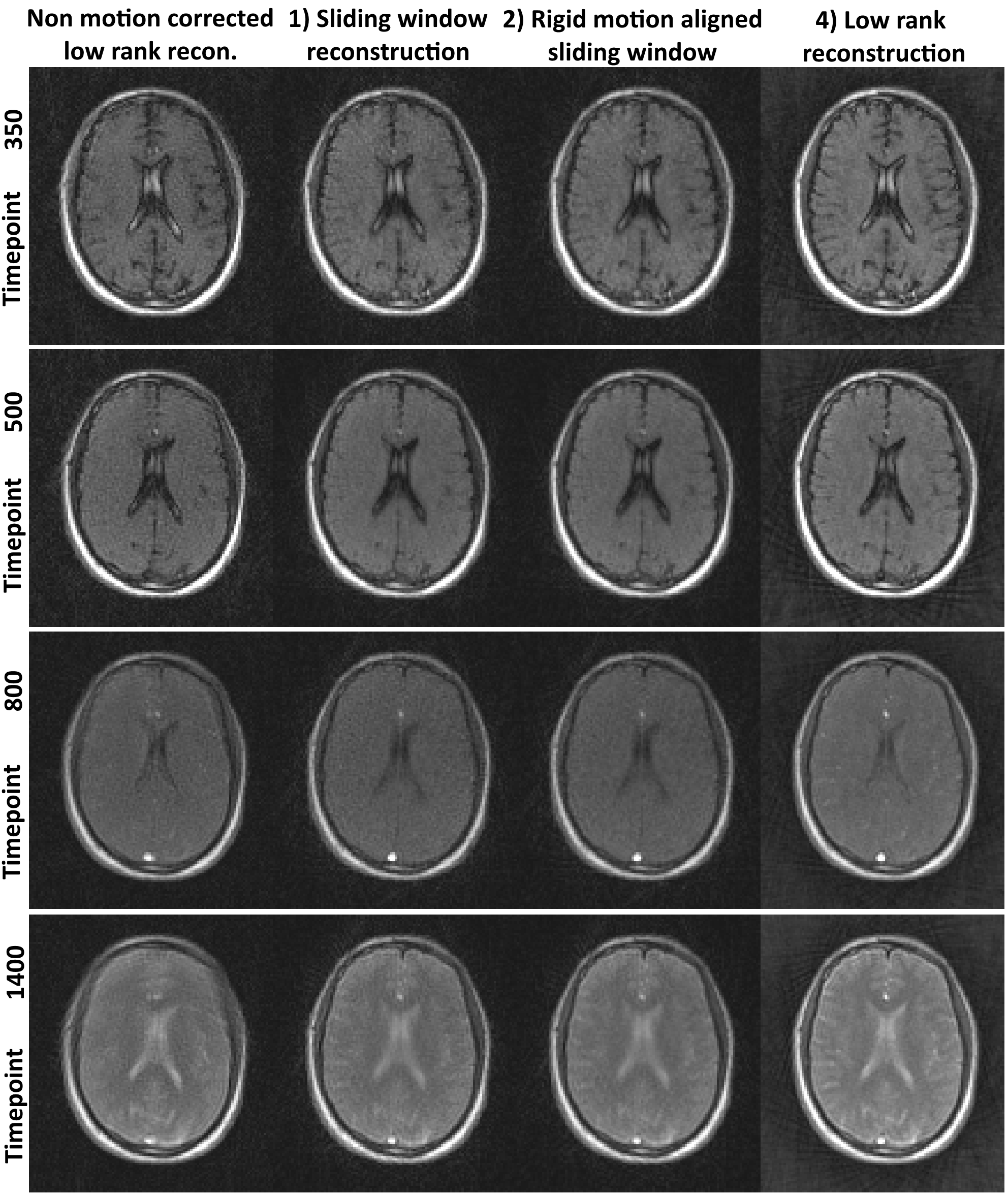


Supporting Information Figure S1: Examples of time-point images for a low rank reconstruction with no motion correction (NMC) and intermediate time-points at different stages of the proposed framework: 1) Sliding window reconstruction, 2) Rigid registration and 4) (motion corrected) Low rank reconstruction.


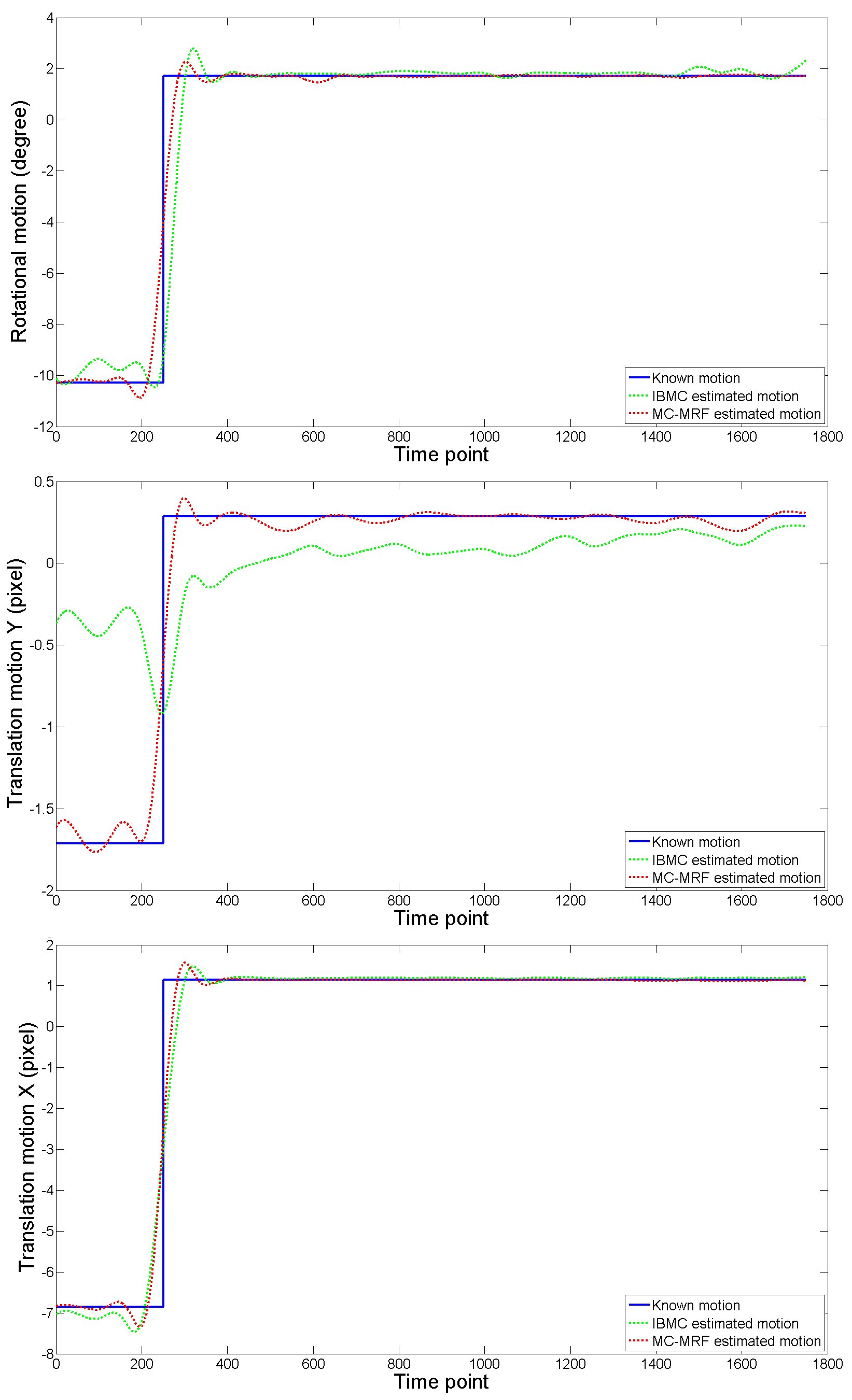


Supporting Information Figure S2: Estimated motion parameters for the simulation experiment with abrupt rigid motion occurring at time-point 250, using image based motion correction (IBMC) and the proposed MC-MRF. Generally, both methods achieve accurate motion estimation, however higher errors are present for IBMC. Both methods present motion estimation errors around the abrupt motion discontinuities.


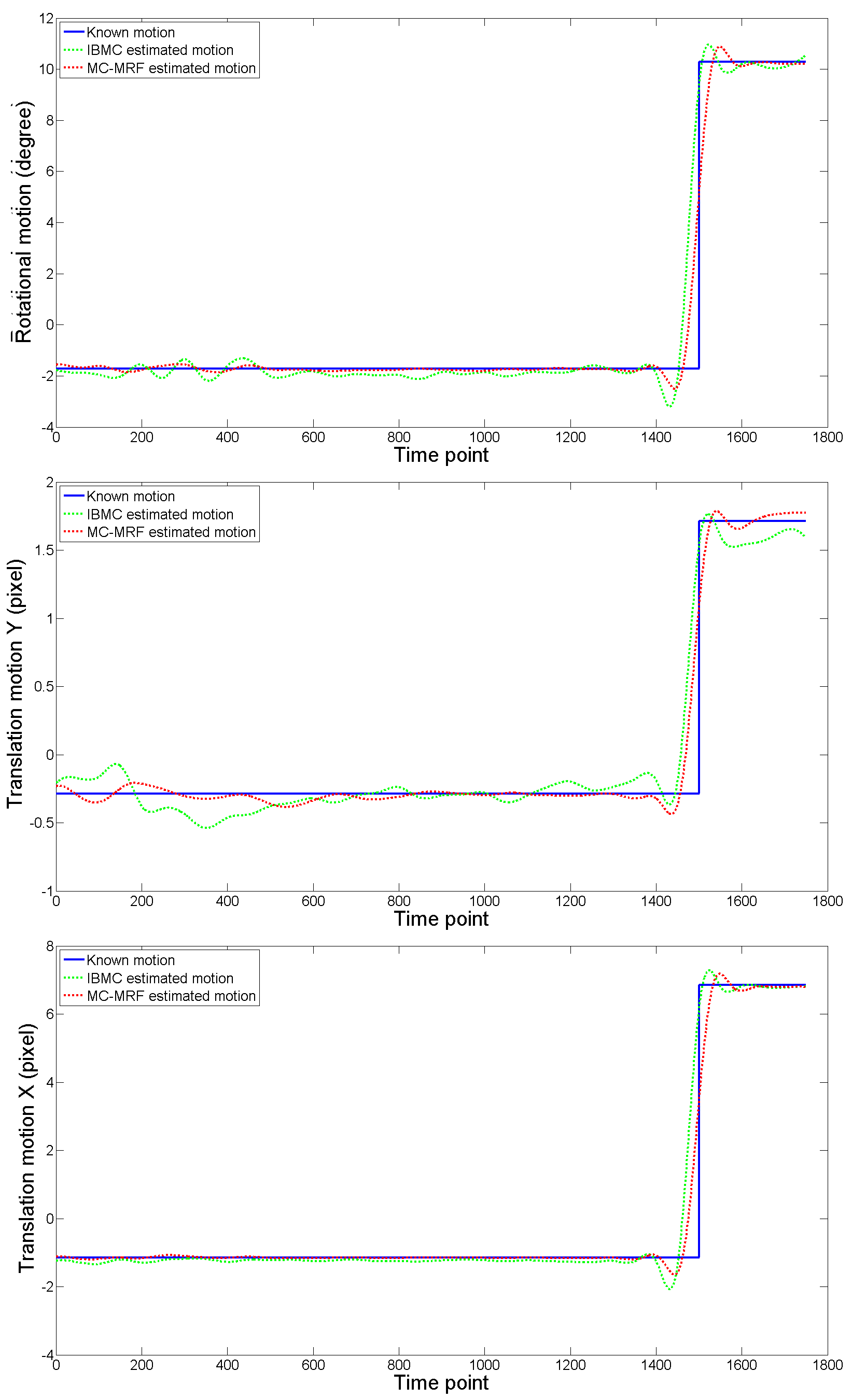


Supporting Information Figure S3: Estimated motion parameters for the simulation experiment with abrupt rigid motion occurring at time-point 1500, using image based motion correction (IBMC) and the proposed MC-MRF. Generally, both methods achieve accurate motion estimation, however higher errors are present for IBMC. Both methods present motion estimation errors around the abrupt motion discontinuities.


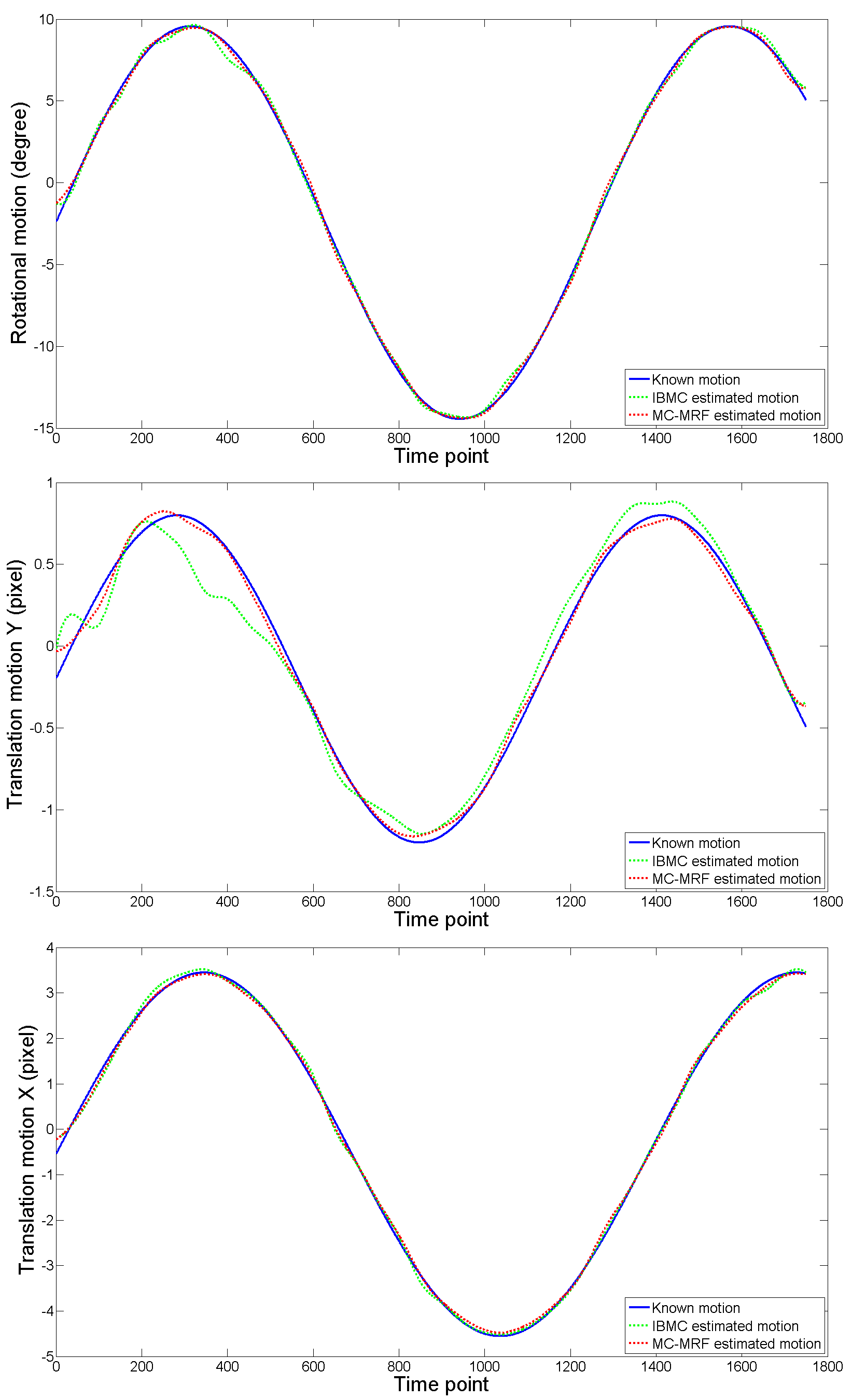


Supporting Information Figure S4: Estimated motion parameters for the simulation experiment with sinusoidally varying motion, using image based motion correction (IBMC) and the proposed MC-MRF. Generally, both methods achieve accurate motion estimation, however higher errors are present for IBMC.


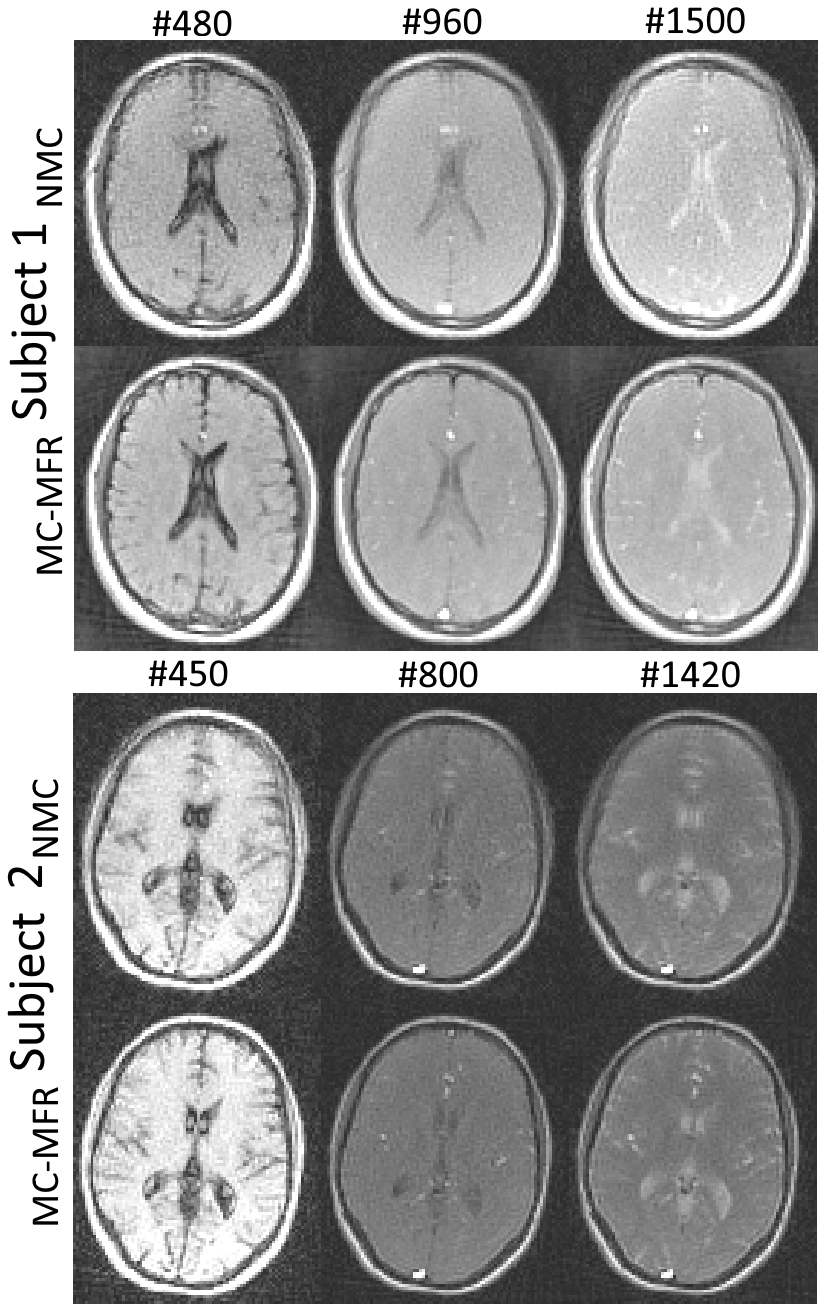


Supporting Information Figure S5: Time-point images for two different subjects with no motion correction (NMC) and the proposed motion corrected MRF (MC-MRF) from an acquisition with in-plane motion. In the presence of motion, low rank reconstruction with no motion correction introduces ghosting and blurring. MC-MRF greatly reduces motion artefacts, revealing image structures otherwise obscured.


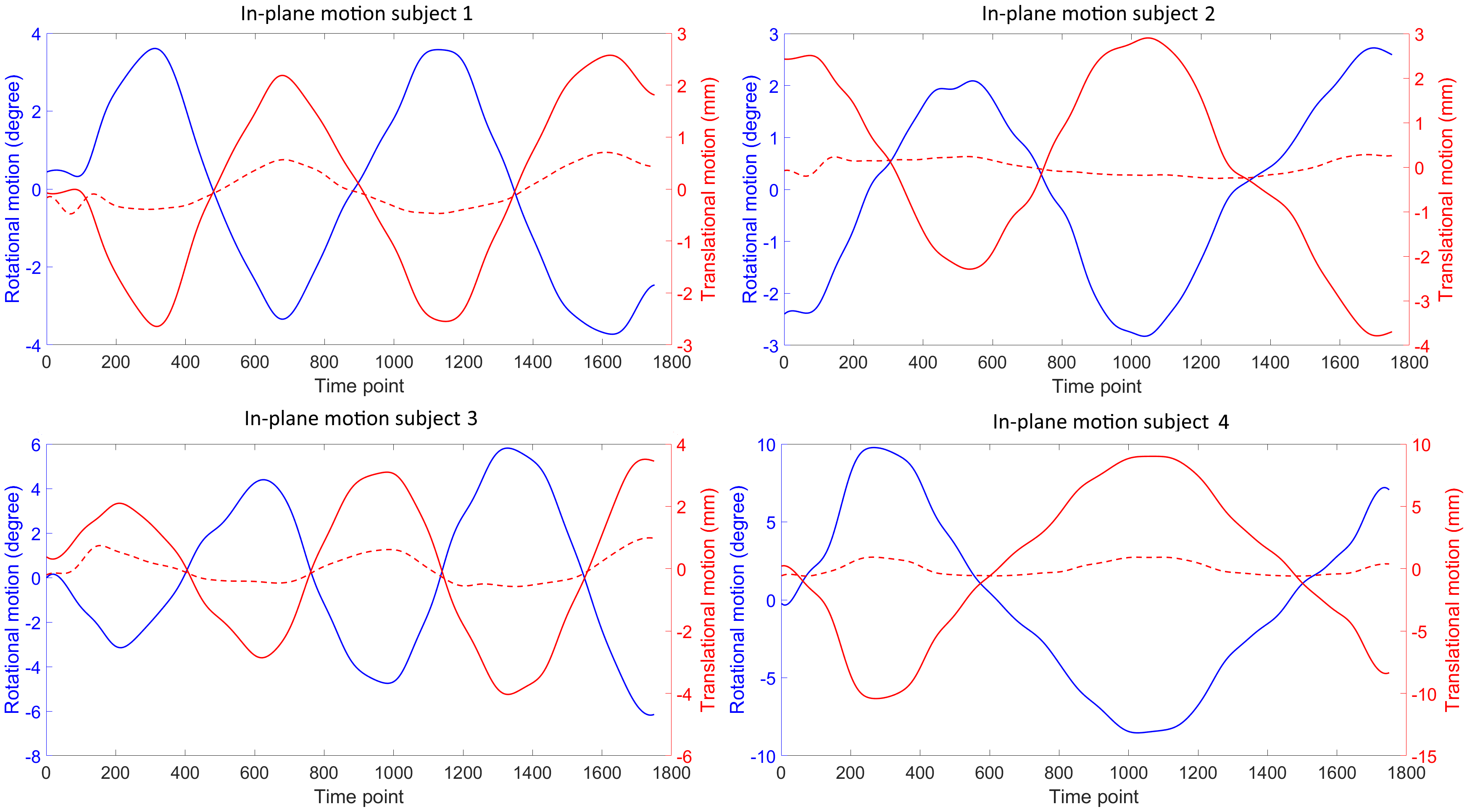


Supporting Information Figure S6. Estimated rigid body motion in four representative brain subject in-vivo scans with in-plane motion. Rotational motion is shown in blue, left-right translation is shown in continuous red and anterior-posterior translation is shown in dashed red. The estimated motion captures the periodic nature of motion is subjects instructed to continuously move during the acquisition. Note different motion amplitudes performed by different subjects.
